# Supplementary material for: The P2X7R-NLRP3 and AIM2 Inflammasome Platforms Mark the Complexity/Severity of Viral or Metabolic Liver Damage
Source: Int J Mol Sci. 2022 Jul 4;23(13):7447. doi: 10.3390/ijms23137447 (PMC9267345; doi:10.3390/ijms23137447)
Supplement: Supplementary file 1 [file ijms-23-07447-s001.zip › ijms-1787733-supplementary.pdf]

Supplementary Table S1. Grading and staging of liver biopsies in patients with NAFLD/NASH according to the Brunt score.

	**NAFLD** (n=10)										**NASH** (n=11)											**P**
Patient	1	2	3	4	5	6	7	8	9	10	11	12	13	14	15	16	17	18	19	20	21	
Grade	1	1	1	1	1	1	1	1	2	1	4	4	4	4	8	4	4	7	4	7	4	<0.001
Stage	1	0	1	1	0	0	1	0	0	0	2	2	4	3	4	3	3	3	4	4	3	<0.001

Supplementary Table S2. Grading and staging of liver biopsies in patients with HCV according to the Ishak score.

	**HCV-nMet** (n=12)												**HCV-Met** (n=13)													**P**
Patient	22	23	24	25	26	27	28	29	30	31	32	33	34	35	36	37	38	39	40	41	42	43	44	45	46	
Grade	7	8	6	7	7	5	6	6	6	7	11	7	9	7	3	5	7	3	10	5	4	8	8	5	9	0.05
Stage	2	5	0	1	3	3	0	0	0	3	5	0	5	5	0	5	4	1	0	3	0	4	0	5	3	0.75

Supplementary Table S3. Gene expression of pro-inflammatory cytokines (TNFα and IL-6) and a fibrotic marker (TGFβ) in liver biopsies

	**NAFLD/NASH**			**HCV**				
	**All** (n=20)	**NAFLD** (n=9)	**NASH** (n=11)	**All** (n=23)	**HCV-nMet** (n=12)	**HCV-Met** (n=11)	**P***	**P****
TNFα	0.52 [0.29-1.13]	0.37 [0.20-0.45]	1.12 [0.52-1.81]	1.43 [0.98-2.33]	1.54 [0.98-3.84][a]	1.43 [0.82-1.89][a]	0.003	0.001
IL-6	2.38 [0.98-3.72]	2.38 [2.03-3.42]	2.13 [0.86-4.72]	6.30 [2.71-11.2]	4.11 [2.00-9.19]	8.72 [3.45-12.4]	0.04	0.14
TGFβ	0.37 [0.20-0.76]	0.23 [0.18-0.45]	0.46 [0.35-1.53]	0.42 [0.33-0.74]	0.37 [0.29-1.61]	0.46 [0.41-0.67]	0.16	0.056

Data are reported as median [range].
* p value for the difference between the two main groups (NAFLD/NASH *vs* HCV).
** p value for the difference between the 4 subgroups (NAFLD, NASH, HCV-nMet, HCV-Met).
[a] $p<0.05$ for the difference with the NAFLD subgroup.

Supplementary Table S4. List of Antibodies

Protein	Antibody Reference	Dilution	Company
P2X7 receptor	APR-004	1:100	Alomone Labs, Jerusalem, Israel
NLRP3	AG-20B-0014	1:200	AdipoGen, San Diego, CA, USA
AIM2	SC-293174	1:50	Santa Cruz Biotech, Dallas, TX, USA

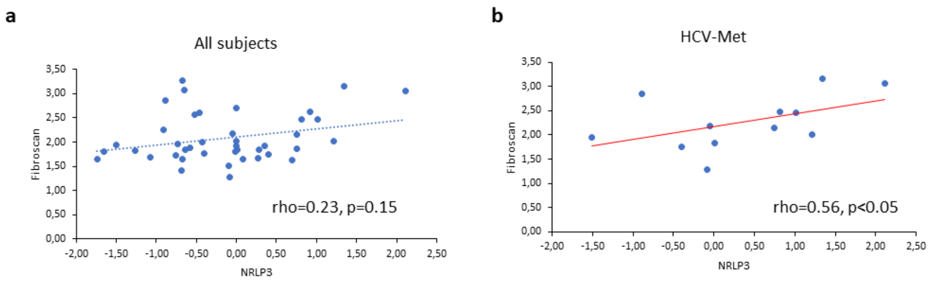

Supplementary Figure S1 Linear relationship between Fibroscan index and liver NLRP3 expression in all subjects (a) and in the HCV-Met subgroup (b).
